# Combined Amino Acid Positron Emission Tomography and Advanced Magnetic Resonance Imaging in Glioma Patients

**DOI:** 10.3390/cancers11020153

**Published:** 2019-01-29

**Authors:** Philipp Lohmann, Jan-Michael Werner, N. Jon Shah, Gereon R. Fink, Karl-Josef Langen, Norbert Galldiks

**Affiliations:** 1 Institute of Neuroscience and Medicine (INM-3, -4, -5, -11), Forschungszentrum Juelich, 52425 Juelich, Germany; n.j.shah@fz-juelich.de (N.J.S.); gereon.fink@uk-koeln.de (G.R.F.); k.j.langen@fz-juelich.de (K.-J.L.); n.galldiks@fz-juelich.de (N.G.); 2 Department of Neurology, Faculty of Medicine and University Hospital Cologne, University of Cologne, 50937 Cologne, Germany; jan-michael.werner@uk-koeln.de; 3 JARA-BRAIN-Translational Medicine, 52074 Aachen, Germany; 4 Department of Neurology, RWTH Aachen University, 52074 Aachen, Germany; 5 Department of Nuclear Medicine, RWTH Aachen University, 52074 Aachen, Germany; 6 Center of Integrated Oncology (CIO), Universities of Cologne and Bonn, 50937 Cologne, Germany

**Keywords:** [^11^C]-methyl-l-methionine (MET), *O*-(2-[^18^F]-fluoroethyl)-l-tyrosine (FET), 3,4-dihydroxy-6-[^18^F]-fluoro-l-phenylalanine (FDOPA), magnetic resonance spectroscopy, perfusion-weighted imaging, diffusion-weighted imaging, chemical exchange saturation transfer, brain tumors, high-grade glioma, hybrid PET/MRI scanner

## Abstract

Imaging techniques such as positron emission tomography (PET) and magnetic resonance imaging (MRI) provide valuable information about brain tumor patients. Particularly amino acid PET, advanced MRI techniques, and combinations thereof are of great interest for the non-invasive assessment of biological characteristics in patients with primary or secondary brain cancer. A methodological innovation that potentially advances research in patients with brain tumors is the increasing availability of hybrid PET/MRI systems, which enables the simultaneous acquisition of both imaging modalities. Furthermore, the advent of ultra-high field MRI scanners operating at magnetic field strengths of 7 T or more will allow further development of metabolic MR imaging at higher resolution. This review focuses on the combination of amino acid PET with MR spectroscopic imaging, perfusion- and diffusion-weighted imaging, as well as chemical exchange saturation transfer in patients with high-grade gliomas, especially glioblastomas.

## 1. Introduction

At present, contrast-enhanced magnetic resonance imaging (MRI) is the method of choice for brain tumor diagnostics since MRI provides excellent soft tissue contrast, comparatively high resolution, and is widely available. On the downside, its specificity for neoplastic tissue is low, hampering the evaluation of tumor extent in both enhancing and non-enhancing gliomas as well as the differentiation of tumor progression from non-specific treatment-related changes [1,2,3].

[^18^F]-2-Fluoro-2-deoxy-D-glucose (FDG) is the most extensively used positron emission tomography (PET) tracer to date and has hence gained exceptional importance in general oncology. In stark contrast, its applicability in brain tumor diagnostics is hindered by high levels of physiological glucose uptake in the cerebral cortex resulting in diminished contrast between tumor and background. On the contrary, the cerebral uptake of radiolabeled amino acids is low while it is typically increased in brain tumors, resulting in an enhanced tumor-to-background contrast [4,5]. Importantly, the uptake of amino acid tracers is independent of the blood-brain barrier integrity, allowing the evaluation of amino acid uptake in non-enhancing gliomas [1,6]. Additionally, amino acid PET has demonstrated its usefulness for the differentiation of tumor progression from treatment-related changes [7,8,9,10,11,12] and for various other indications, e.g., treatment monitoring, prognostication or tumor grading [4,5,6,13,14,15,16,17]. Among amino acid tracers labelled with carbon-11 used for PET imaging in neuro-oncology, [^11^C]-methyl-L-methionine (MET) is currently best evaluated [18,19]. However, the short half-life of carbon-11 (20 min) limits its use to centers with an on-site cyclotron. To overcome such logistical limitations, amino acid tracers have been labeled with fluorine-18, which has a longer half-life of 110 min [4,6]. For that reason, O-(2-[^18^F]-fluoroethyl)-L-tyrosine (FET) has replaced MET in many neuro-oncological centers. Subsequently, FET has become the most widely used radiotracer for brain tumor diagnostics, especially in Western Europe. Another fluorine-18 labeled amino acid tracer is 3,4-dihydroxy-6-[^18^F]-fluoro-L-phenylalanine (FDOPA), originally developed to evaluate the dopamine synthesis in the basal ganglia, which also shows increased uptake in brain tumors [20,21]. Although brain tumors distant to the striatum are depicted with high contrast, the high striatal uptake of FDOPA limits its usefulness for tumor delineation especially near the basal ganglia [22,23]. The Response Assessment in Neuro-Oncology (RANO) working group considers the additional value of amino acid PET for brain tumor diagnostics as highly relevant and recommends its use at every stage of brain tumor management [24].

The combination of MRI and PET, especially if hybrid PET/MR scanners are used, offers great potential for brain tumor diagnostics. This technology is particularly attractive when ultra-high field MRI scanners operating at magnetic field strengths of 7 T or more are widely available, allowing comparative anatomical and metabolic imaging at high resolution. Currently, amino acid PET tracers are the best evaluated and most frequently used PET tracers for brain tumor diagnostics. Therefore, this review focuses on the combination of amino acid PET with MR spectroscopy (MRS), perfusion- and diffusion-weighted imaging (PWI, DWI), and chemical exchange saturation transfer (CEST) in patients with glioblastoma (GBM).

## 2. Search Strategy

A PubMed search of the published literature with the combination of the search terms “glioblastoma”, “brain tumors”, “high-grade glioma”, “positron emission tomography”, “magnetic resonance imaging”, “magnetic resonance spectroscopy”, “perfusion-weighted imaging”, “diffusion-weighted imaging”, “chemical exchange saturation transfer”, “PET”, “amino acid PET”, “MRI”, “advanced MRI”, “MRS”, “PWI”, “DWI”, “CEST”, and “hybrid PET/MR” before and inclusive of December 2018 was performed. Additional literature was retrieved from the reference lists of all identified articles. Furthermore, articles identified through searches of the authors’ files were included. Only papers in English were considered.

## 3. Amino Acid PET and MRS

MR spectroscopy (MRS) is a non-invasive method to detect selected water-soluble metabolites in vivo. In magnetic resonance, an externally applied magnetic field experienced by the nucleus is modulated by the distribution of surrounding electrons. Given that every molecule presents with a slightly different distribution of electrons, every nucleus experiences a slightly different magnetic field.

Consequently, every molecule has its characteristic magnetic field ‘signature’ that, combined with interactions amongst the nuclei, results in slightly different resonance frequencies leading to differential signals. These signal differences are used in MRS to identify the metabolites of interest. Every nucleus possessing a non-zero spin can be theoretically used for MRS, e.g., protons, carbon-13 and phosphorous-31. However, due to the low abundance of the latter and limited resolution of metabolite profiles in vivo, mainly proton spectra are used in clinical practice.

Metabolites that are frequently assessed by MRS are lactate, lipids, alanine, N-acetylaspartate (NAA), glutamine, glutamate, 2-hydroxyglutarate (2-HG), citrate, creatine, choline, and myo-inositol. In gliomas, with increasing grade of malignancy, NAA and creatine are usually decreased, whereas choline, lipids and lactate are increased [25,26,27]. Furthermore, the accumulation of 2-HG caused by gene mutations encoding for the enzyme isocitrate dehydrogenase (IDH) can be detected by 2-HG spectroscopy at 3 T [28]. Although several studies demonstrated the feasibility of 2-HG spectroscopy in a clinical setting [29,30,31,32], 2-HG spectroscopy remains challenging due to a small and complex signal and it is therefore not yet established in clinical routine [33].

Two different MRS methods exist using either data from a single voxel or multiple voxels in a single slice or multiple slices of the investigated organ. Single-voxel spectroscopy is the most widely used method due to simple data acquisition and relatively short scanning time. Moreover, the comparatively high signal-to-noise ratio achieved by single-voxel spectroscopy results in high-quality spectra enabling a quantitative analysis and straightforward interpretation. However, the manually preselected region of interest (ROI) based on information from T2-weighted or contrast-enhanced MRI may represent only a fraction of the tumor, potentially resulting in an incomplete evaluation of tumor biology.

Multi-voxel spectroscopy techniques may overcome these limitations by covering a more extended two- or three-dimensional ROI at higher spatial resolution. Especially in heterogenous lesions, this allows detection of subtle intratumoral signal alterations [34]. Another advantage of smaller voxels used in multi-voxel spectroscopy is the reduction of partial volume effects as structures such as cerebrospinal fluid or fat may diminish the quality of spectra and can be excluded from ROI definition. However, because of the difficulty of achieving good enough shimming over a large enough region, multi-voxel spectroscopy can have a lower signal-to-noise ratio, a reduced spectral quality for individual voxels, requires longer scanning times, and is technically more demanding than single-voxel spectroscopy [35].

In a few studies, MRS combined with amino acid PET in glioma patients was used. D´Souza and colleagues [36] evaluated in 29 high-grade glioma patients the diagnostic performance of MET PET and single-voxel MRS at 3 T for the correct diagnosis of tumor recurrence after neurooncological treatment including radiotherapy and chemotherapy. Metrics derived from both MET PET (i.e., tumor/brain ratios) and single-voxel MRS (i.e., choline/creatine ratios) suggested a high diagnostic accuracy of 85–90% for the detection of high-grade glioma recurrence. Floeth and co-workers [37] explored the use of combined FET PET and single-voxel MRS at 1.5 T in 50 patients with newly diagnosed lesions suspicious for gliomas on contrast-enhanced MRI. Based on the neuropathological assessment, gliomas could be identified with an accuracy of 68% using conventional MRI alone. Accuracy could be increased to 97% when conventional MR imaging was used in combination with FET PET and single-voxel MRS. In that study, FET lesion/brain ratios and NAA/choline ratios were identified as significant independent predictors for the identification of glioma tissue.

Stadlbauer et al. [38] used two-dimensional multi-voxel spectroscopy at 1.5 T in combination with FET PET to spatially correlate concentrations of choline, creatine, and NAA with FET uptake in 15 glioma patients. The authors found significant correlations between increased FET uptake and parameters derived from MRS. More precisely, increased FET uptake was significantly correlated with the extent of neuronal loss (NAA) and partially with membrane proliferation (choline), indicating that both PET and MRS provide complementary information. Yet, the number of patients included in that study was relatively small and two-dimensional MRS allowed only limited coverage of the tumor, especially in comparison with amino acid PET, which provided three-dimensional information concerning tracer distribution.

**Figure 1:**
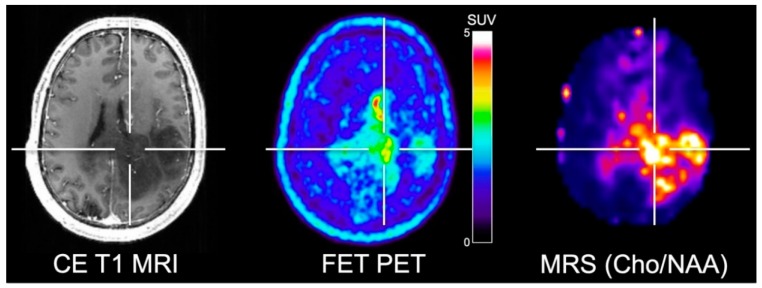
Simultaneously acquired contrast-enhanced (CE) T1-weighted MRI, FET PET and whole-brain MRS shows elevated choline/N-acetylaspartate (Cho/NAA) ratios in a patient with neuropathologically confirmed glioblastoma. Areas of contrast enhancement, FET uptake and increased Cho/NAA are spatially incongruent. Image courtesy of Joerg Mauler, Institute of Neuroscience and Medicine, Forschungszentrum Juelich, Germany, and Andrew Maudsley, Department of Radiology, University of Miami Medical School, Miami, FL, USA.

To overcome these limitations, Mauler and colleagues [39] investigated the spatial correlation between FET PET and three-dimensional MRS covering the whole brain. Forty-one glioma patients were investigated using a 3 T hybrid PET/MR scanner. Importantly, the authors reported that the FET uptake and increased choline/NAA ratios were not consistently spatially congruent (Figure 1). Unfortunately, however, no spatial correlation with neuropathological information obtained via stereotactic biopsy was performed to further explore the differences between amino acid uptake and the metabolites detected by MRS in gliomas.

## 4. Amino Acid PET and PWI

PWI is a non-invasive MRI technique to measure blood flow quantitatively. In Neuro-Oncology, the parameters relative cerebral blood volume (rCBV) and cerebral blood flow (rCBF) are frequently assessed. Most commonly, a gadolinium-based contrast agent is used to assess tissue perfusion. After i.v. injection, the passage of the contrast agent leads to i) a local magnetic field distortion (susceptibility effect) in the vicinity of the vessels causing a signal drop in T2- or T2*-weighted MRI, also called dynamic susceptibility contrast (DSC), or ii) a shortening of T1-relaxation time causing a signal increase in T1-weighted MRI, also called dynamic contrast-enhanced (DCE) MRI. While DSC data requires only the first pass of intravascular contrast agent for assessing tissue perfusion, DCE additionally evaluates information about the continuous accumulation of the contrast agent in the extracellular space. Consequently, the acquisition times for DCE are longer than for DSC.

Another PWI method called arterial spin labelling (ASL) does not require a contrast agent. Here, endogenous water molecules in blood vessels are magnetically labeled by applying a specific radiofrequency pulse. Passage of these labeled molecules through the tissue of interest leads to a reduction of signal intensity in proportion to the perfusion. However, by dispensing with the administration of a contrast agent, the signal-to-noise ratio of ASL is inherently low, so that repetitive signal averaging is mandatory resulting in prolonged acquisition times. ASL significantly benefits from higher magnetic field strengths. Thus, ultra-high field MRI scanners operating at magnetic field strengths of up to 7 T may boost its clinical utility in the future.

**Figure 2:**
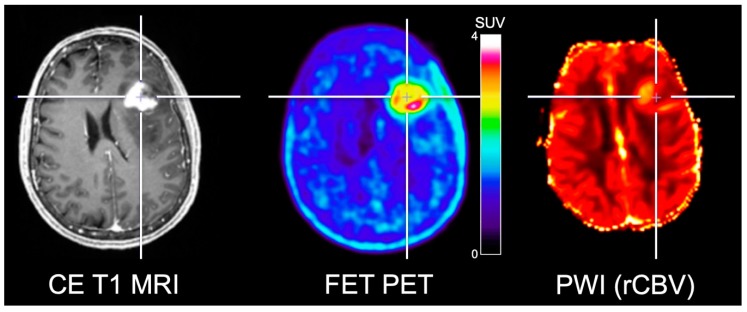
Simultaneously acquired contrast-enhanced (CE) T1-weighted MRI, FET PET and relative cerebral blood volume (rCBV) obtained from PWI in a patient with neuropathologically confirmed glioblastoma. FET PET identifies metabolically active tumor areas without an increased rCBV. Image courtesy of Christian Filss, Institute of Neuroscience and Medicine, Forschungszentrum Juelich, Juelich, Germany.

Several studies combined PWI and amino acid PET in patients with high-grade gliomas for the delineation of the glioma extent. Filss and colleagues [40] compared DSC PWI and FET PET acquired concurrently using a 3 T hybrid PET/MR scanner in 56 glioma patients (24 patients with GBM). One main finding was that FET PET tumor volumes were significantly larger than tumor volumes delineated by rCBV maps. Furthermore, the spatial congruence of the two methods was poor, and the localization of tumor hotspots identified by PWI and FET PET yielded inconsistent results indicating that the delineation of glioma extent is not appropriately reflected by PWI and hence cannot replace amino acid PET (Figure 2). These findings were confirmed subsequently [41,42]. Besides, similar results were reported by Cicone and co-workers for the comparison of DSC PWI at 1.5 T and FDOPA PET [22].

Verger et al. [43] investigated the usefulness of FET PET and DSC PWI at 3 T for the grading of gliomas in 72 patients with newly diagnosed glioma. The diagnostic accuracy for glioma grading was comparable for both FET PET and rCBV with an area under the receiver-operating characteristic curve of about 0.80. However, neuropathological diagnoses were not based on the revised WHO classification from 2016 [44], which limits the applicability of the results. The authors also found in 78% of patients a considerable spatial disparity between the local hotspots delineated using the two methods, consistent with previous studies [22,40,41].

Dandois and co-workers [45] compared the diagnostic accuracy of DSC PWI at 1.5 T and amino acid PET using MET for the differentiation of recurrent glioma from radiation necrosis in 28 high-grade glioma patients. The diagnostic performance was comparable between rCBV maps and MET PET for this clinically relevant question. These findings were reinforced by other studies [36,46,47]. However, in the majority of the cases, the diagnosis of either recurrent glioma or radiation necrosis was based on clinical observation and radiological follow-up rather than neuropathology. In stark contrast, Verger and colleagues [48] reported that FET PET seems to be superior to PWI at 3 T to diagnose recurrent glioma. Importantly, and in contrast to the latter studies mentioned above, the definite diagnosis was based on neuropathology in about 80% of the cases. These findings were confirmed by another study [49]. Subsequently, Roodakker et al. [50] compared regional MET PET and PWI at 3 T with local neuropathology in patients with en bloc-resected oligodendrogliomas. They showed that MET uptake correlated with tumor cell density throughout the entire tumor volume in all patients. Interestingly, tumor perfusion (rCBV) did not correlate with MET uptake or with any other histological marker.

Morana and colleagues [51] compared FDOPA PET and ASL at 1.5 T for the grading and prediction of tumor progression in 26 pediatric gliomas. Authors reported a better diagnostic performance for FDOPA PET with an area under the receiver-operating characteristic curve of 0.95 compared to 0.88 for rCBF derived from ASL.

The reported differences across the studies highlight the need for prospective studies with larger cohorts and neuropathological validation to further evaluate the differences between perfusion MRI and amino acid PET. Furthermore, a more recent meta-analysis regarding PWI in high-grade gliomas by Patel and co-workers [52] revealed a considerable heterogeneity of applied MR sequences, acquisition and post-processing parameters utilized in PWI studies of brain tumors thereby hampering comparability and reproducibility of results.

## 5. Amino Acid PET and DWI

Diffusion-weighted imaging (DWI) is an MRI technique based on the measurement of Brownian motion of water molecules to generate image contrast. DWI contrast uses two opposing gradient pulses; the first one induces a phase shift in water molecules, leading to a signal reduction. Subsequently, a second opposed gradient pulse is applied, which rephases the water molecules in the region of interest, leading to a recovery of the water signal. If water molecules moved out of the region of interest within the time between the two pulses, the number of water molecules contributing to the water signal is reduced. Therefore, the signal intensity is decreased. By variation of the amplitude, pulse duration, and time between the pulses, the degree of diffusion-weighting, represented by the b-value, can be altered. The apparent diffusion coefficient (ADC) is calculated from different b-values (typical range of b-values, 0–1000 s/mm^2^), and represents a quantitative measure of diffusivity [53]. In brain tumors, hypointense signals in ADC maps (indicating lower ADC values) are suspicious for neoplastic tissue due to high tumor cellularity leading to a restriction of diffusivity [6].

**Figure 3:**
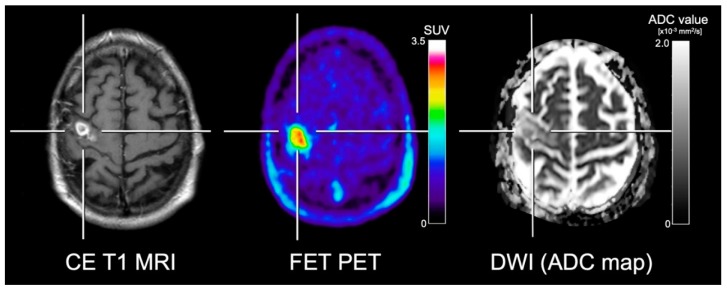
Contrast-enhanced (CE) T1-weighted MRI, FET PET and the apparent diffusion coefficient (ADC) map obtained from DWI in a patient with neuropathologically confirmed glioblastoma. In contrast to the ADC map, FET PET shows the metabolically active tumor tissue with high contrast. Furthermore, the ADC map shows slightly increased diffusivity in various subregions, erroneously indicating treatment-related changes.

Several studies addressed the spatial congruency of DWI and amino acid PET in glioma patients with mixed results. Karavaeva and colleagues [54] demonstrated a significant spatial relationship of FDOPA uptake and ADC signal alterations at 1.5 T in patients with recurrent high-grade glioma. Furthermore, a significant spatial correlation of both ADC values and FDOPA uptake with the cellular proliferation rate was reported. However, it remains unclear whether the cellular proliferation rate was associated with tumor progression or treatment-related tissue reactions such as inflammatory processes. On the other hand, several other studies reported contradictory results concerning the spatial congruency of amino acid uptake and ADC abnormalities [55,56,57]. Recently, Popp and co-workers [57] showed that tumor volumes in patients with recurrent glioblastoma delineated using ADC maps acquired at 1.5 T were frequently localized outside of areas with increased FET uptake. Another study by Kinoshita [58] and colleagues reported that MET PET demonstrated a more robust and reliable estimation of cell density in gliomas compared to ADC maps acquired at 3 T. These controversial study results concerning the spatial congruency are most likely related to the fact that amino acid PET and DWI encode different biological properties [57,59] (Figure 3).

Pyka and colleagues [49] compared FET PET and DWI at 3 T for the differentiation of tumor progression from treatment-related changes in 47 high-grade glioma patients. The authors reported a superior performance for FET PET compared to ADC (AUC, 0.86 vs. 0.69). The combination of FET PET and ADC with other MRI metrics such as rCBV resulted in an AUC improvement to 0.89.

It should be pointed out that DWI has a low spatial resolution and a poor signal-to-noise ratio. Although stronger gradients and higher magnetic field strengths can increase signal-to-noise ratios, susceptibility artifacts would become concurrently more pronounced. Another limitation of DWI is the compromised reproducibility and comparability of ADC values, even when the same MR scanner is used [60]. Moreover, diffusivity can also be altered by ischemia, infection, inflammation, gliosis, necrosis, or vascular proliferation, suggesting non-specificity of DWI changes [6,61,62].

## 6. Amino Acid PET and CEST

Chemical exchange saturation transfer (CEST) is an MRI technique for the indirect detection of different metabolites using the MR free water signal [63]. CEST contrasts are based on the spontaneous exchange of free water protons and protons bound to metabolites (solute-bound protons). After saturation of solute-bound protons by the application of a radio-frequency pulse with a specific frequency depending on the metabolite of interest, these protons exchange with free water protons and cause a signal reduction of free water. Thus, the CEST signal depends upon the exchange rate between free water and solute-bound protons and the concentration of the metabolite of interest [64,65].

CEST has already been applied for the measurement of glycogen (GlycoCEST) [66], glucose (GlucoCEST) [67], glutamate (GluCEST) [68], amide proton transfer (APT) [69], and intracellular pH [69,70,71]. The signal used to generate CEST contrasts is complex and small. Therefore, CEST imaging may benefit from higher magnetic field strengths (> 3 T) due to an increased signal-to-noise ratio and spectral dispersion. However, complex data processing steps are required to evaluate metabolite concentrations from CEST contrasts [72,73,74,75].

Although several studies evaluated CEST in patients with GBM [75,76,77], only few studies directly compared amino acid PET and CEST in more than 5 patients. Da Silva et al. [78] compared FET PET and APT CEST MRI in 8 high-grade gliomas patients using a 3 T hybrid PET/MR scanner. The authors showed that FET PET and APT CEST are spatially incongruent and reflect different biological information. However, although CEST and amino acid PET were acquired simultaneously, the number of patients was low, and no correlation with local neuropathology was obtained.

## 7. Conclusions and Outlook

The combination of amino acid PET and advanced MRI methods offers a variety of new vistas for the assessment of brain tumors with the potential to overcome the limitations of conventional MRI. Amino acid PET and advanced MRI methods already provided promising results for the clinical management of patients with GBM. Both the complementary and additive value of these methods as well as their incongruent findings, suggesting differential biological information, warrant further investigation including neuropathological validation. The increasing advent of hybrid PET/MR scanners offers the great potential of comparative studies using both amino acid PET and advanced MRI in a single session. Furthermore, the clinical benefits of simultaneous PET/MR imaging need to be balanced against the relatively high cost of such an approach. Of note, the increasing availability of ultra-high field MRI scanners with magnetic field strengths of 7 T will help to develop novel techniques thereby promoting advanced MRI in neuro-oncology as almost all contrasts benefit from the higher spatial resolution related to the increased signal-to-noise ratio.

Amino acid PET is a robust and attractive approach for clinicians for many reasons including easy scan reading. Furthermore, most studies using amino acid PET in neuro-oncology provide comparable results across different scanners, which is also a consequence of national and international efforts concerning the standardization of amino acid PET acquisition and evaluation in brain tumor imaging [24,79]. Recently, joint practice guidelines were developed by the European Association of Nuclear Medicine (EANM), the Society of Nuclear Medicine and Molecular Imaging (SNMMI), the European Association of Neuro-Oncology (EANO), and the working group for Response Assessment in Neuro-Oncology with PET (PET-RANO) [80].

In contrast, the yet missing standardization of advanced MRI methods is—at least in part—likely to account for conflicting data. In a significant number of advanced MRI studies, self-developed or self-optimized MR sequence protocols and acquisition parameters, as well as post-processing tools, were used, thereby impeding comparability and reproducibility of the results. Besides, vendors of MRI and PET scanners should strive for further standardization of imaging protocols and data processing since better comparability and easier reproducibility of the data is likely to boost their clinical value. Finally, further implementation of various advanced MRI methods as well as amino acid PET in clinical routine requires the validation of neuroimaging findings through neuropathology.

To conclude, advanced MRI in combination with amino acid PET has the potential to become an essential diagnostic tool for improving the clinical management of patients with high-grade gliomas, as well as in light of emerging high-throughput analysis methods of large-scale data sets such as radiomics and machine learning [16,81,82].

## References

[1] Lohmann P., Stavrinou P., Lipke K., Bauer E.K., Ceccon G., Werner J.M., Neumaier B., Fink G.R., Shah N.J., Langen K.J. (2018). FET PET reveals considerable spatial differences in tumour burden compared to conventional MRI in newly diagnosed glioblastoma. Eur. J. Nucl. Med. Mol. Imaging.

[2] Dhermain F.G., Hau P., Lanfermann H., Jacobs A.H., van den Bent M.J. (2010). Advanced MRI and PET imaging for assessment of treatment response in patients with gliomas. Lancet Neurol..

[3] Ahluwalia M.S., Wen P.Y. (2011). Antiangiogenic therapy for patients with glioblastoma: Current challenges in imaging and future directions. Expert Rev. Anticancer Ther..

[4] Herholz K. (2017). Brain Tumors: An Update on Clinical PET Research in Gliomas. Semin. Nucl. Med..

[5] Galldiks N., Langen K.J., Pope W.B. (2015). From the clinician’s point of view—What is the status quo of positron emission tomography in patients with brain tumors?. Neuro Oncol..

[6] Langen K.J., Galldiks N., Hattingen E., Shah N.J. (2017). Advances in neuro-oncology imaging. Nat. Rev. Neurol..

[7] Galldiks N., Dunkl V., Stoffels G., Hutterer M., Rapp M., Sabel M., Reifenberger G., Kebir S., Dorn F., Blau T. (2015). Diagnosis of pseudoprogression in patients with glioblastoma using *O*-(2-[18F]fluoroethyl)-l-tyrosine PET. Eur. J. Nucl. Med. Mol. Imaging.

[8] Belliveau J.G., Bauman G., Macdonald D.R. (2016). Detecting tumor progression in glioma: Current standards and new techniques. Expert Rev. Anticancer Ther..

[9] Galldiks N., Stoffels G., Filss C., Rapp M., Blau T., Tscherpel C., Ceccon G., Dunkl V., Weinzierl M., Stoffel M. (2015). The use of dynamic *O*-(2-18F-fluoroethyl)-l-tyrosine PET in the diagnosis of patients with progressive and recurrent glioma. Neuro Oncol..

[10] Ceccon G., Lohmann P., Stoffels G., Judov N., Filss C.P., Rapp M., Bauer E., Hamisch C., Ruge M.I., Kocher M. (2017). Dynamic *O*-(2-18F-fluoroethyl)-l-tyrosine positron emission tomography differentiates brain metastasis recurrence from radiation injury after radiotherapy. Neuro Oncol..

[11] Galldiks N., Kocher M., Langen K.-J. (2017). Pseudoprogression after glioma therapy: An update. Expert Rev. Neurother..

[12] Muoio B., Giovanella L., Treglia G. (2018). Recent developments of 18F-FET PET in neuro-oncology. Curr. Med. Chem..

[13] Suchorska B., Albert N.L., Tonn J.C. (2018). Role of amino-tracer PET for decision-making in neuro-oncology. Curr. Opin. Neurol..

[14] Langen K.J., Galldiks N. (2018). Update on amino acid PET of brain tumours. Curr. Opin. Neurol..

[15] Galldiks N., Law I., Pope W.B., Arbizu J., Langen K.J. (2017). The use of amino acid PET and conventional MRI for monitoring of brain tumor therapy. Neuroimage Clin..

[16] Lohmann P., Kocher M., Steger J., Galldiks N. (2018). Radiomics derived from amino-acid PET and conventional MRI in patients with high-grade gliomas. Q J. Nucl. Med. Mol. Imaging.

[17] Galldiks N., Albert N.L., Sommerauer M., Grosu A.L., Ganswindt U., Law I., Preusser M., Le Rhun E., Vogelbaum M.A., Zadeh G. (2017). PET imaging in patients with meningioma-report of the RANO/PET Group. Neuro Oncol..

[18] Singhal T., Narayanan T.K., Jain V., Mukherjee J., Mantil J. (2008). 11C-l-methionine positron emission tomography in the clinical management of cerebral gliomas. Mol. Imaging Biol..

[19] Galldiks N., Ullrich R., Schroeter M., Fink G.R., Jacobs A.H., Kracht L.W. (2010). Volumetry of [(11)C]-methionine PET uptake and MRI contrast enhancement in patients with recurrent glioblastoma multiforme. Eur. J. Nucl. Med. Mol. Imaging.

[20] Becherer A., Karanikas G., Szabo M., Zettinig G., Asenbaum S., Marosi C., Henk C., Wunderbaldinger P., Czech T., Wadsak W. (2003). Brain tumour imaging with PET: A comparison between [18F]fluorodopa and [11C]methionine. Eur. J. Nucl. Med. Mol. Imaging.

[21] Herrmann K., Czernin J., Cloughesy T., Lai A., Pomykala K.L., Benz M.R., Buck A.K., Phelps M.E., Chen W. (2014). Comparison of visual and semiquantitative analysis of 18F-FDOPA-PET/CT for recurrence detection in glioblastoma patients. Neuro Oncol..

[22] Cicone F., Filss C.P., Minniti G., Rossi-Espagnet C., Papa A., Scaringi C., Galldiks N., Bozzao A., Shah N.J., Scopinaro F. (2015). Volumetric assessment of recurrent or progressive gliomas: Comparison between F-DOPA PET and perfusion-weighted MRI. Eur. J. Nucl. Med. Mol. Imaging.

[23] Galldiks N., Langen K.J. (2015). Applications of PET imaging of neurological tumors with radiolabeled amino acids. Q J. Nucl. Med. Mol. Imaging.

[24] Albert N.L., Weller M., Suchorska B., Galldiks N., Soffietti R., Kim M.M., la Fougere C., Pope W., Law I., Arbizu J. (2016). Response Assessment in Neuro-Oncology working group and European Association for Neuro-Oncology recommendations for the clinical use of PET imaging in gliomas. Neuro Oncol..

[25] Negendank W.G., Sauter R., Brown T.R., Evelhoch J.L., Falini A., Gotsis E.D., Heerschap A., Kamada K., Lee B.C., Mengeot M.M. (1996). Proton magnetic resonance spectroscopy in patients with glial tumors: A multicenter study. J. Neurosurg..

[26] Glunde K., Bhujwalla Z.M., Ronen S.M. (2011). Choline metabolism in malignant transformation. Nat. Rev. Cancer.

[27] Herminghaus S., Pilatus U., Moller-Hartmann W., Raab P., Lanfermann H., Schlote W., Zanella F.E. (2002). Increased choline levels coincide with enhanced proliferative activity of human neuroepithelial brain tumors. NMR Biomed..

[28] Choi C., Ganji S.K., DeBerardinis R.J., Hatanpaa K.J., Rakheja D., Kovacs Z., Yang X.L., Mashimo T., Raisanen J.M., Marin-Valencia I. (2012). 2-hydroxyglutarate detection by magnetic resonance spectroscopy in IDH-mutated patients with gliomas. Nat. Med..

[29] Natsumeda M., Igarashi H., Nomura T., Ogura R., Tsukamoto Y., Kobayashi T., Aoki H., Okamoto K., Kakita A., Takahashi H. (2014). Accumulation of 2-hydroxyglutarate in gliomas correlates with survival: A study by 3.0-tesla magnetic resonance spectroscopy. Acta Neuropathol. Commun..

[30] Andronesi O.C., Loebel F., Bogner W., Marjanska M., Vander Heiden M.G., Iafrate A.J., Dietrich J., Batchelor T.T., Gerstner E.R., Kaelin W.G. (2016). Treatment Response Assessment in IDH-Mutant Glioma Patients by Noninvasive 3D Functional Spectroscopic Mapping of 2-Hydroxyglutarate. Clin. Cancer Res..

[31] De la Fuente M.I., Young R.J., Rubel J., Rosenblum M., Tisnado J., Briggs S., Arevalo-Perez J., Cross J.R., Campos C., Straley K. (2016). Integration of 2-hydroxyglutarate-proton magnetic resonance spectroscopy into clinical practice for disease monitoring in isocitrate dehydrogenase-mutant glioma. Neuro Oncol..

[32] Choi C., Raisanen J.M., Ganji S.K., Zhang S., McNeil S.S., An Z., Madan A., Hatanpaa K.J., Vemireddy V., Sheppard C.A. (2016). Prospective Longitudinal Analysis of 2-Hydroxyglutarate Magnetic Resonance Spectroscopy Identifies Broad Clinical Utility for the Management of Patients With IDH-Mutant Glioma. J. Clin. Oncol..

[33] Andronesi O.C., Rapalino O., Gerstner E., Chi A., Batchelor T.T., Cahill D.P., Sorensen A.G., Rosen B.R. (2013). Detection of oncogenic IDH1 mutations using magnetic resonance spectroscopy of 2-hydroxyglutarate. J. Clin. Investig..

[34] Nelson S.J. (2003). Multivoxel magnetic resonance spectroscopy of brain tumors. Mol. Cancer Ther..

[35] Rabinov J.D., Lee P.L., Barker F.G., Louis D.N., Harsh G.R., Cosgrove G.R., Chiocca E.A., Thornton A.F., Loeffler J.S., Henson J.W. (2002). In vivo 3-T MR spectroscopy in the distinction of recurrent glioma versus radiation effects: Initial experience. Radiology.

[36] D’Souza M.M., Sharma R., Jaimini A., Panwar P., Saw S., Kaur P., Mondal A., Mishra A., Tripathi R.P. (2014). 11C-MET PET/CT and advanced MRI in the evaluation of tumor recurrence in high-grade gliomas. Clin. Nucl. Med..

[37] Floeth F.W., Pauleit D., Wittsack H.J., Langen K.J., Reifenberger G., Hamacher K., Messing-Junger M., Zilles K., Weber F., Stummer W. (2005). Multimodal metabolic imaging of cerebral gliomas: Positron emission tomography with [18F]fluoroethyl-l-tyrosine and magnetic resonance spectroscopy. J. Neurosurg..

[38] Stadlbauer A., Prante O., Nimsky C., Salomonowitz E., Buchfelder M., Kuwert T., Linke R., Ganslandt O. (2008). Metabolic imaging of cerebral gliomas: Spatial correlation of changes in *O*-(2-18F-fluoroethyl)-l-tyrosine PET and proton magnetic resonance spectroscopic imaging. J. Nucl. Med..

[39] Mauler J., Maudsley A.A., Langen K.J., Nikoubashman O., Stoffels G., Sheriff S., Lohmann P., Filss C., Galldiks N., Kops E.R. (2018). Spatial Relationship of Glioma Volume Derived from (18)F-FET PET and Volumetric MR Spectroscopy Imaging: A Hybrid PET/MRI Study. J. Nucl. Med..

[40] Filss C.P., Galldiks N., Stoffels G., Sabel M., Wittsack H.J., Turowski B., Antoch G., Zhang K., Fink G.R., Coenen H.H. (2014). Comparison of 18F-FET PET and perfusion-weighted MR imaging: A PET/MR imaging hybrid study in patients with brain tumors. J. Nucl. Med..

[41] Henriksen O.M., Larsen V.A., Muhic A., Hansen A.E., Larsson H.B., Poulsen H.S., Law I. (2016). Simultaneous evaluation of brain tumour metabolism, structure and blood volume using [(18)F]-fluoroethyltyrosine (FET) PET/MRI: Feasibility, agreement and initial experience. Eur. J. Nucl. Med. Mol. Imaging.

[42] Gottler J., Lukas M., Kluge A., Kaczmarz S., Gempt J., Ringel F., Mustafa M., Meyer B., Zimmer C., Schwaiger M. (2017). Intra-lesional spatial correlation of static and dynamic FET-PET parameters with MRI-based cerebral blood volume in patients with untreated glioma. Eur. J. Nucl. Med. Mol. Imaging.

[43] Verger A., Filss C.P., Lohmann P., Stoffels G., Sabel M., Wittsack H.J., Kops E.R., Galldiks N., Fink G.R., Shah N.J. (2017). Comparison of (18)F-FET PET and perfusion-weighted MRI for glioma grading: A hybrid PET/MR study. Eur. J. Nucl. Med. Mol. Imaging.

[44] Louis D.N., Perry A., Reifenberger G., von Deimling A., Figarella-Branger D., Cavenee W.K., Ohgaki H., Wiestler O.D., Kleihues P., Ellison D.W. (2016). The 2016 World Health Organization Classification of Tumors of the Central Nervous System: A summary. Acta Neuropathol..

[45] Dandois V., Rommel D., Renard L., Jamart J., Cosnard G. (2010). Substitution of 11C-methionine PET by perfusion MRI during the follow-up of treated high-grade gliomas: Preliminary results in clinical practice. J. Neuroradiol..

[46] Kim Y.H., Oh S.W., Lim Y.J., Park C.K., Lee S.H., Kang K.W., Jung H.W., Chang K.H. (2010). Differentiating radiation necrosis from tumor recurrence in high-grade gliomas: Assessing the efficacy of 18F-FDG PET, 11C-methionine PET and perfusion MRI. Clin. Neurol. Neurosurg..

[47] Jena A., Taneja S., Gambhir A., Mishra A.K., D’Souza M.M., Verma S.M., Hazari P.P., Negi P., Jhadav G.K. (2016). Glioma Recurrence Versus Radiation Necrosis: Single-Session Multiparametric Approach Using Simultaneous *O*-(2-18F-Fluoroethyl)-l-Tyrosine PET/MRI. Clin. Nucl. Med..

[48] Verger A., Filss C.P., Lohmann P., Stoffels G., Sabel M., Wittsack H.J., Kops E.R., Galldiks N., Fink G.R., Shah N.J. (2018). Comparison of *O*-(2-(18)F-Fluoroethyl)-l-Tyrosine Positron Emission Tomography and Perfusion-Weighted Magnetic Resonance Imaging in the Diagnosis of Patients with Progressive and Recurrent Glioma: A Hybrid Positron Emission Tomography/Magnetic Resonance Study. World Neurosurg..

[49] Pyka T., Hiob D., Preibisch C., Gempt J., Wiestler B., Schlegel J., Straube C., Zimmer C. (2018). Diagnosis of glioma recurrence using multiparametric dynamic 18F-fluoroethyl-tyrosine PET-MRI. Eur. J. Radiol..

[50] Roodakker K.R., Alhuseinalkhudhur A., Al-Jaff M., Georganaki M., Zetterling M., Berntsson S.G., Danfors T., Strand R., Edqvist P.H., Dimberg A. (2018). Region-by-region analysis of PET, MRI, and histology in en bloc-resected oligodendrogliomas reveals intra-tumoral heterogeneity. Eur. J. Nucl. Med. Mol. Imaging.

[51] Morana G., Piccardo A., Tortora D., Puntoni M., Severino M., Nozza P., Ravegnani M., Consales A., Mascelli S., Raso A. (2017). Grading and outcome prediction of pediatric diffuse astrocytic tumors with diffusion and arterial spin labeling perfusion MRI in comparison with 18F-DOPA PET. Eur. J. Nucl. Med. Mol. Imaging.

[52] Patel P., Baradaran H., Delgado D., Askin G., Christos P., John Tsiouris A., Gupta A. (2017). MR perfusion-weighted imaging in the evaluation of high-grade gliomas after treatment: A systematic review and meta-analysis. Neuro Oncol..

[53] Koh D.M., Padhani A.R. (2006). Diffusion-weighted MRI: A new functional clinical technique for tumour imaging. Br. J. Radiol..

[54] Karavaeva E., Harris R.J., Leu K., Shabihkhani M., Yong W.H., Pope W.B., Lai A., Nghiemphu P.L., Liau L.M., Chen W. (2015). Relationship Between [18F]FDOPA PET Uptake, Apparent Diffusion Coefficient (ADC), and Proliferation Rate in Recurrent Malignant Gliomas. Mol. Imaging Biol..

[55] Rose S., Fay M., Thomas P., Bourgeat P., Dowson N., Salvado O., Gal Y., Coulthard A., Crozier S. (2013). Correlation of MRI-derived apparent diffusion coefficients in newly diagnosed gliomas with [18F]-fluoro-l-dopa PET: What are we really measuring with minimum ADC?. AJNR Am. J. Neuroradiol..

[56] Choi H., Paeng J.C., Cheon G.J., Park C.K., Choi S.H., Min H.S., Kang K.W., Chung J.K., Kim E.E., Lee D.S. (2014). Correlation of 11C-methionine PET and diffusion-weighted MRI: Is there a complementary diagnostic role for gliomas?. Nucl. Med. Commun..

[57] Popp I., Bott S., Mix M., Oehlke O., Schimek-Jasch T., Nieder C., Nestle U., Bock M., Yuh W.T.C., Meyer P.T. (2018). Diffusion-weighted MRI and ADC versus FET-PET and GdT1w-MRI for gross tumor volume (GTV) delineation in re-irradiation of recurrent glioblastoma. Radiother. Oncol..

[58] Kinoshita M., Arita H., Okita Y., Kagawa N., Kishima H., Hashimoto N., Tanaka H., Watanabe Y., Shimosegawa E., Hatazawa J. (2016). Comparison of diffusion tensor imaging and (11)C-methionine positron emission tomography for reliable prediction of tumor cell density in gliomas. J. Neurosurg..

[59] Tietze A., Boldsen J.K., Mouridsen K., Ribe L., Dyve S., Cortnum S., Ostergaard L., Borghammer P. (2015). Spatial distribution of malignant tissue in gliomas: Correlations of 11C-l-methionine positron emission tomography and perfusion- and diffusion-weighted magnetic resonance imaging. Acta Radiol..

[60] Sasaki M., Yamada K., Watanabe Y., Matsui M., Ida M., Fujiwara S., Shibata E., Acute Stroke Imaging Standardization Group-Japan I. (2008). Variability in absolute apparent diffusion coefficient values across different platforms may be substantial: A multivendor, multi-institutional comparison study. Radiology.

[61] Huang R.Y., Neagu M.R., Reardon D.A., Wen P.Y. (2015). Pitfalls in the neuroimaging of glioblastoma in the era of antiangiogenic and immuno/targeted therapy—Detecting illusive disease, defining response. Front. Neurol..

[62] Sundgren P.C., Fan X., Weybright P., Welsh R.C., Carlos R.C., Petrou M., McKeever P.E., Chenevert T.L. (2006). Differentiation of recurrent brain tumor versus radiation injury using diffusion tensor imaging in patients with new contrast-enhancing lesions. Magn. Reson. Imaging.

[63] Wolff S.D., Balaban R.S. (1990). NMR imaging of labile proton exchange. J. Magn. Reson..

[64] Ward K.M., Aletras A.H., Balaban R.S. (2000). A new class of contrast agents for MRI based on proton chemical exchange dependent saturation transfer (CEST). J. Magn. Reson..

[65] Ward K.M., Balaban R.S. (2000). Determination of pH using water protons and chemical exchange dependent saturation transfer (CEST). Magn. Reson. Med..

[66] Van Zijl P.C., Jones C.K., Ren J., Malloy C.R., Sherry A.D. (2007). MRI detection of glycogen in vivo by using chemical exchange saturation transfer imaging (glycoCEST). Proc. Natl. Acad. Sci. USA.

[67] Walker-Samuel S., Ramasawmy R., Torrealdea F., Rega M., Rajkumar V., Johnson S.P., Richardson S., Goncalves M., Parkes H.G., Arstad E. (2013). In vivo imaging of glucose uptake and metabolism in tumors. Nat. Med..

[68] Cai K., Haris M., Singh A., Kogan F., Greenberg J.H., Hariharan H., Detre J.A., Reddy R. (2012). Magnetic resonance imaging of glutamate. Nat. Med..

[69] Zhou J., Modo M., Bulte J.W.M. (2011). Amide Proton Transfer Imaging of the Human Brain.

[70] Zhou J., Payen J.F., Wilson D.A., Traystman R.J., van Zijl P.C. (2003). Using the amide proton signals of intracellular proteins and peptides to detect pH effects in MRI. Nat. Med..

[71] Jones C.K., Schlosser M.J., van Zijl P.C., Pomper M.G., Golay X., Zhou J. (2006). Amide proton transfer imaging of human brain tumors at 3T. Magn. Reson. Med..

[72] Van Zijl P.C., Yadav N.N. (2011). Chemical exchange saturation transfer (CEST): What is in a name and what isn’t?. Magn. Reson. Med..

[73] Wu B., Warnock G., Zaiss M., Lin C., Chen M., Zhou Z., Mu L., Nanz D., Tuura R., Delso G. (2016). An overview of CEST MRI for non-MR physicists. EJNMMI Phys..

[74] Zaiss M., Bachert P. (2013). Chemical exchange saturation transfer (CEST) and MR Z-spectroscopy in vivo: A review of theoretical approaches and methods. Phys. Med. Biol..

[75] Dreher C., Oberhollenzer J., Meissner J.E., Windschuh J., Schuenke P., Regnery S., Sahm F., Bickelhaupt S., Bendszus M., Wick W. (2018). Chemical exchange saturation transfer (CEST) signal intensity at 7T MRI of WHO IV degrees gliomas is dependent on the anatomic location. J. Magn. Reson. Imaging.

[76] Regnery S., Adeberg S., Dreher C., Oberhollenzer J., Meissner J.E., Goerke S., Windschuh J., Deike-Hofmann K., Bickelhaupt S., Zaiss M. (2018). Chemical exchange saturation transfer MRI serves as predictor of early progression in glioblastoma patients. Oncotarget.

[77] Sagiyama K., Mashimo T., Togao O., Vemireddy V., Hatanpaa K.J., Maher E.A., Mickey B.E., Pan E., Sherry A.D., Bachoo R.M. (2014). In vivo chemical exchange saturation transfer imaging allows early detection of a therapeutic response in glioblastoma. Proc. Natl. Acad. Sci. USA.

[78] Da Silva N.A., Lohmann P., Fairney J., Magill A.W., Oros Peusquens A.M., Choi C.H., Stirnberg R., Stoffels G., Galldiks N., Golay X. (2018). Hybrid MR-PET of brain tumours using amino acid PET and chemical exchange saturation transfer MRI. Eur. J. Nucl. Med. Mol. Imaging.

[79] Vander Borght T., Asenbaum S., Bartenstein P., Halldin C., Kapucu O., Van Laere K., Varrone A., Tatsch K., European Association of Nuclear Medicine (EANM) (2006). EANM procedure guidelines for brain tumour imaging using labelled amino acid analogues. Eur. J. Nucl. Med. Mol. Imaging.

[80] Law I., Albert N.L., Arbizu J., Boellaard R., Drzezga A., Galldiks N., la Fougere C., Langen K.J., Lopci E., Lowe V. (2018). Joint EANM/EANO/RANO practice guidelines/SNMMI procedure standards for imaging of gliomas using PET with radiolabelled amino acids and [(18)F]FDG: Version 1.0. Eur. J. Nucl. Med. Mol. Imaging.

[81] Lambin P., Rios-Velazquez E., Leijenaar R., Carvalho S., Van Stiphout R.G.P.M., Granton P., Zegers C.M.L., Gillies R., Boellard R., Dekker A. (2012). Radiomics: Extracting more information from medical images using advanced feature analysis. Eur. J. Cancer.

[82] Lohmann P., Kocher M., Ceccon G., Bauer E.K., Stoffels G., Viswanathan S., Ruge M.I., Neumaier B., Shah N.J., Fink G.R. (2018). Combined FET PET/MRI radiomics differentiates radiation injury from recurrent brain metastasis. Neuroimage Clin..

